# Individualized arterial spin labeling background suppression by rapid T1 mapping during acquisition

**DOI:** 10.1007/s00330-022-08550-8

**Published:** 2022-02-04

**Authors:** T. Lindner, H. Guerreiro, F. Austein, J. Fiehler

**Affiliations:** Department of Diagnostic and Interventional Neuroradiology, University Hospital Hamburg-Eppendorf, Martinistr. 52, Gebauede W14, 20251 Hamburg, Germany

**Keywords:** Perfusion, Healthy volunteers, Arteries

## Abstract

**Objective:**

Arterial spin labeling blood perfusion signal relies on the difference between a label and a control image. Background suppression pulses are commonly used to improve the contrast, yet these are based on estimates of tissue relaxation times. The aim of this study is to improve the perfusion contrast by individualizing the timing of these background suppression pulses by means of T1 mapping.

**Methods:**

The optimized timing of the background suppression pulses is obtained by rapid T1 mapping employing the variable flip angle technique. Ten healthy volunteers were included in this study. To compare the results, visual grading and the Wilcoxon signed-rank test was used comparing three categories of image quality.

**Results:**

The readers confirmed that the images of the proposed method generally show a higher signal-to-background ratio and cortical structures are better visible. Noise was mostly comparable to the standard method. Relative blood flow was statistically significant higher in the modified method.

**Conclusion:**

The individually optimized background suppression pulses improve the image appearance and allow for a better visualization of cortical structures. The proposed technique however prolongs scan time, which can be seen as negative result, yet needs to be further evaluated.

**Key Points:**

• *Background suppression timing in ASL can vary.*

• *Both the label and control condition can be modified for T1 mapping.*

• *Adapting the pulse timing improves the signal-to-background ratio.*

## Introduction

Arterial spin labeling (ASL) is a known method for non-contrast-enhanced perfusion imaging mainly used in the brain and offers possibilities to obtain reliable information about underlying pathologies that influence perfusion [1]. Compared to contrast agent-enhanced perfusion imaging, ASL is a more complex sequence that relies on exact timing of gradient and radiofrequency (RF) pulses. The currently recommended ASL technique is pseudo-continuous ASL (PCASL). This sequence generally consists of the following parts: First, the image volume is saturated; then, the magnetization of the water protons of arterial blood flowing through the labeling plane in the neck is being either inverted (label) or not (control), followed by a waiting period denoted as post labeling delay (PLD) to allow for the blood to reach the tissue and undergo perfusion. During labeling and PLD, two or more background suppression pulses are commonly used which aim for increasing the signal difference between blood perfusion signal and static tissue [1–3]. These are inversion pulses timed in a sense that at the time of image acquisition brain tissue (gray and white matter and cerebrospinal fluid) is being maximally suppressed while blood signal is at maximum. This process is performed either empirically or by using optimization algorithms [4]. However, the input values to calculate the background suppression times are often taken from the literature, not reflecting patient individual deviations from these values. The T1 relaxation times of gray matter can vary up to 10% from patient to patient [5, 6]. Thus, individual background suppression pulses optimizing blood and tissue difference signal appear attractive to improve the visualization in ASL imaging. Since relaxation times of tissues are generally obtained by T1 mapping, adding this technique to an ASL protocol can provide the specific relaxation times. T1 mapping however is a time-consuming method since image acquisition has to be performed individually at multiple time points after inversion per time point [7]. Well-known methods use Look-Locker readout or the modified Look-Locker (MOLLI) technique, which are however still too long to justify its use during a routine scan protocol [8, 9]. Recently, a method was presented that calculated the T1 values from the M0 scan [10]. Another technique that was introduced several years ago employs two different flip angles to get a good estimate of the T1 relaxation times in short scan times [11–13]. The present study aims to employ this variable flip angle (vfa) method to provide rapid T1 mapping of gray matter during ASL imaging and in further consequence to adapt the background suppression pulse timing to potentially improve the visual impression of the scans.

## Materials and methods

The test collective consisted of 10 healthy volunteers (4 women, 6 men, mean age 26.8 years, range: 22–41 years). All subjects underwent scanning on a 3-T Siemens MAGNETOM Prisma scanner equipped with a 64-channel head coil. The study was approved by the local ethical committee; volunteers gave written informed consent. PCASL scan parameters included the following: 2000-ms labeling duration and 1700-ms post labeling delay, 3D GraSE readout with 3.6 × 3.6 × 4-mm^3^ resolution, TR/TE: 4000/12.06ms. In this study, the label and control acquisitions were different to regular ASL imaging. One acquisition was performed with a single label/control pair with a flip angle (FA) of 9° for label and 20° for control and one was acquired with the FAs switched (i.e., 20° FA for control and 9° FA for label). Acquisition of such a label/control pair took 1:12 min. More details can be found in Table 1. In these acquisitions, no background suppression pulses were used. T1 maps were obtained by the vfa post-processing module using qMRLAB [14]. Then, segmentation of gray and white matter and CSF was performed using SPM12. Multiplying the gray matter mask with the resulting T1 map, a whole-brain mean value of gray matter T1 was calculated and then used for optimum background suppression timing. The regular ASL acquisition had 4 label/control pairs (all with a FA of 20°). Scan time was 4:48 min. This ASL scan was performed twice. Once with the routine fixed background suppression pulse timings and once with adapted. The final images were evaluated by two readers (reader 1: H.G. and reader 2: F.A. with 7 and 8 years of experience in neuroradiology respectively). The readers were presented a standard and a modified image as pair without knowing which is the modified image. The rating was performed with a 3-point grading scale:

−1 = original is better than modified
- 0 = equal
- 1 = modified is better than original.

**Table 1:** Detailed acquisition parameters of the ASL sequence. *PCASL*, pseudo-continuous arterial spin labeling; *GraSE*, gradient spin echo

Labeling technique	PCASL
TR/TE	4000/12.06 ms
Labeling duration	2000 ms
Post labeling delay	1700 ms
Acquisition flip angle	20°
Readout type	3D GraSE
Spatial resolution (in-plane)	3.6 × 3.6 mm^2^
Slice thickness	4mm
Scan time	4:48 min

The rating was done in three categories:

1. Amount of noise in the image
2. Visualization of cortical structures vs. white matter
3. Severity of artifacts

Relative CBF (rCBF) was compared using the Wilcoxon signed-rank test.

## Results

**Fig. 1:**
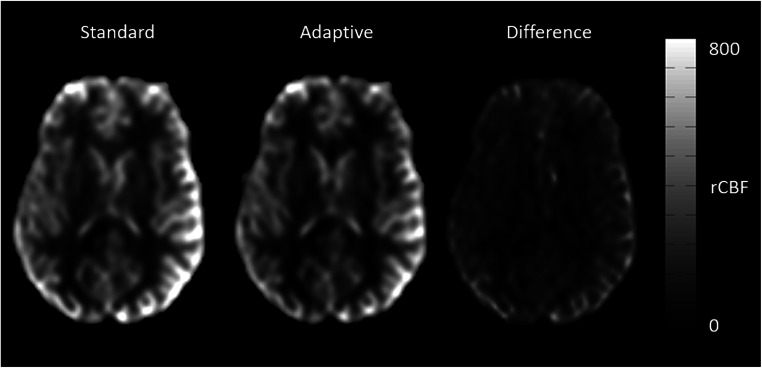
Comparison of standard and adaptive background suppression in the rCBF images. Note the improved gray matter perfusion-to-background difference. The difference image in the right column shows the areas of largest differences

**Fig. 2:**
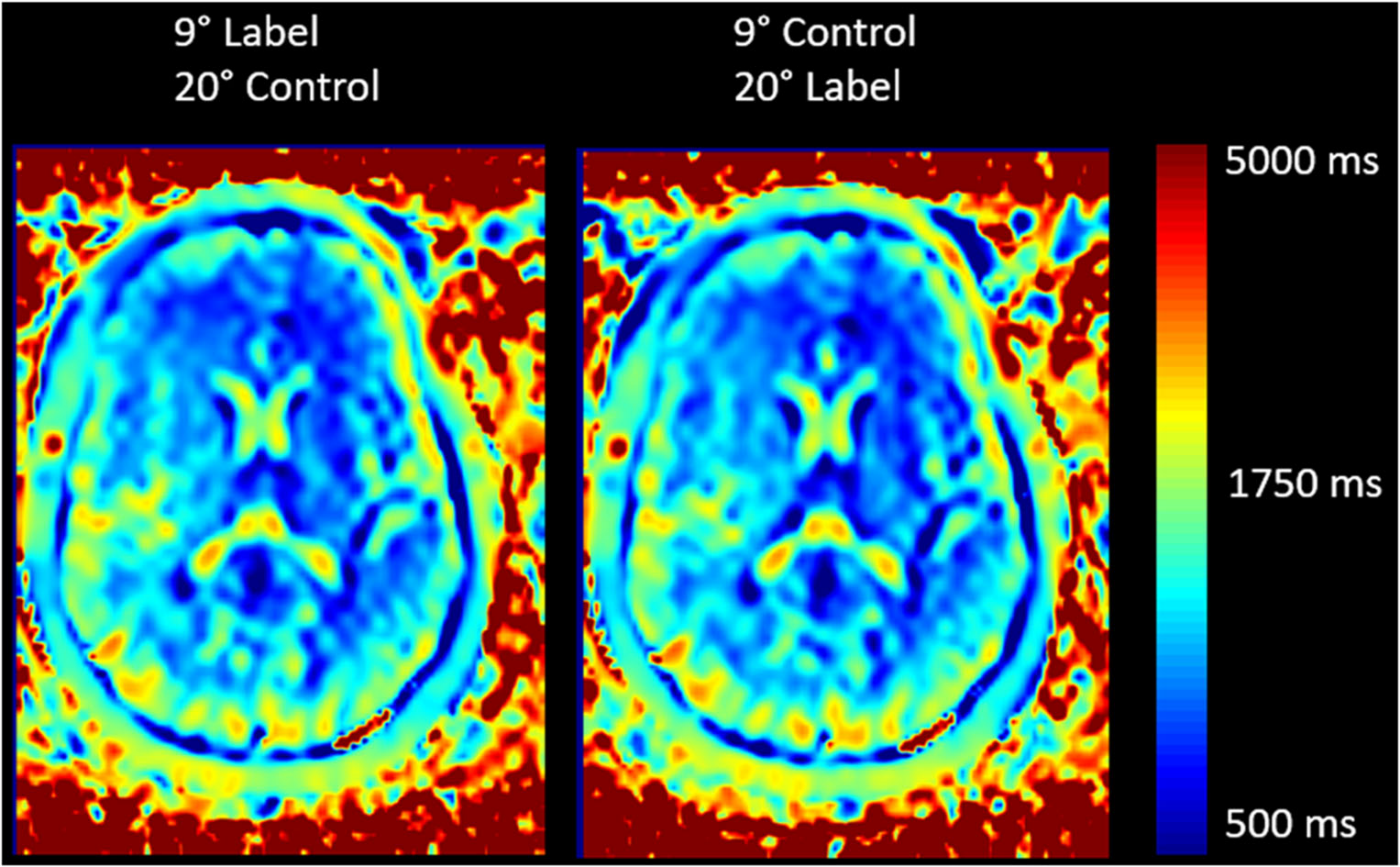
Exemplary T1 map obtained by the variable flip angle method of one volunteer shows that the combination of a high and low flip angle is irrespective of the ASL condition

Adapting the background suppression pulses to the individual values shows higher signal-to-background ratio as compared to a fixed (standard) setting of the background suppression pulses (Fig. 1). This was confirmed by the readers except for artifacts, which are shown to be the same in most cases, but worse in two cases (Table 2). Overall, the ratings were better for the presented method, especially for reader 1 with 8 cases better, 1 same, and 1 worse than the standard method for noise and half of the cases better regarding cortical structure visualization, the rest being equal. Reader 2 also rated 1 case worse regarding noise, 6 equal, and 4 better and for cortical structures the same numbers. Table 3 shows the variability in T1 relaxation times of the volunteers which also shows a deviation of gray matter T1 relaxation times of 12.4% in gray and 6.6% in white matter, being concise with the literature [5]. There was no difference to be observed when the modified flip angle image was acquired as control or label image, i.e., the label type had no influence on the measured T1 values of the tissue (Fig. 2). When measuring gray matter rCBF, an increased signal with *p* < 0.005 could be observed, indicating a better signal-to-background ratio (Table 4).

**Table 2:** Rating of both readers showing that the modified background suppression has been rated better than the original fixed pulse timing

Image	Amount of noise		Visualization of cortical structures		Artifacts	
	Reader 1	Reader 2	Reader 1	Reader 2	Reader 1	Reader 2
1	1	1	0	1	−1	0
2	1	1	1	1	0	0
3	0	0	0	0	0	0
4	1	1	0	1	0	0
5	−1	1	1	1	0	0
6	1	0	0	0	0	0
7	1	0	1	0	0	0
8	1	−1	1	−1	0	−1
9	1	0	1	0	0	0
10	1	0	0	0	0	0
Sum	7	3	5	3	−1	−1

**Table 3:** Mean values of whole-brain T1 relaxation times (in ms). *CSF values might not be accurate due to the long relaxation times

Volunteer	Gray matter	White matter	CSF*
1	1250	897	4096
2	1527	829	4228
3	1331	832	4077
4	1592	996	4196
5	1823	974	4030
6	1497	915	4028
7	1479	859	3906
8	1388	967	3898
9	1255	858	3907
10	1286	875	4063
Mean	1442.8	900.2	4042.9
Std. Dev.	179.7	60.7	115.8

**Table 4:** Mean values of rCBF, Wilcoxon signed-rank test showed a statistically significant difference with *p* < 0.005

Volunteer	rCBF regular	rCBF adaptive
1	613	622
2	519	540
3	543	557
4	448	467
5	473	489
6	625	637
7	557	580
8	521	528
9	600	608
10	681	694
Mean	558.0	572.2
Std. Dev.	72.1	70.0

## Discussion

In this study, we present a method to obtain T1 maps from ASL data by adding one label/control pair with a differing FA to the sequence. Using a low and high FA, it is possible to get an estimate of the gray matter T1 relaxation time, which had a deviation of over 10% in our collective which is consistent with values published in the literature [5, 10]. Such optimizations improve the image quality by reflecting the patient-specific situation rather than fixed values taken from the literature.

In ASL, the desired image contrast is comparably low. In a single label/control pair, only 1–2% of signal are different; thus, strategies are needed to increase the image contrast [1]. This can be done either by increasing the number of repetitions and therefore increasing overall scan time and by means of background suppression [1–3]. Both methods are commonly used. The latter has been investigated by changing the number of pulses, i.e., two pulses provide a less efficient suppression compared to four pulses. However, four pulses reduce image signal due to imperfect inversion. No patient-specific optimization regarding the timing of the pulses has been performed yet and values to calculate the timing have usually been taken from the literature. To make the process easy, the reported average values have been taken into account. Looking into the literature on T1 mapping of human brain tissues reveals a deviation of T1 times up to 10% between the participants of these studies [5].

To obtain T1 values of tissues, several methods have been invented and established. These include inversion-recovery imaging with varying TEs and the Look-Locker technique (and its modifications, e.g., MOLLI) [8, 9]. An alternative is imaging of two individual FAs to estimate relaxation times at otherwise unchanged imaging parameters. This vfa method is faster than the aforementioned techniques and thus seems attractive to be included into a scan protocol.

In this study, both the label and control condition have been acquired once with a differing FA compared to the regular imaging FA to check whether there is any influence of the condition. No changes could be seen in the maps; thus, acquiring one label or control condition with different FA is possible (Fig. 2). From the perspective of implementing this method into the scanner software, this might arguably be the easiest way since only one FA has to be modified and the background suppression disabled while the sequence remains otherwise unchanged. The vfa method in this study showed a deviation of 12.4% in gray matter but only 6.6% in white matter T1 relaxation times, being comparable to values in the literature [5].

The raters overall agreed that the modified strategy shows better results than the original one. There are however cases in which opposite ratings have been performed which can be attributed to various reasons given the subjectivity of ratings. A potential reason is that the order of image pairs was randomized; thus, one rater could have been tired receiving certain cases late in the list.

Post-processing of the data can be automatized when performed directly on the scanner. First, calculating T1 maps from the vfa images, then segmentation of tissues, and finally creating an average value for T1 can be performed within a few seconds since no data transfer is needed; thus, there will be only little time penalty for the optimization process itself.

A potential interesting field of use is pediatric neuroradiology since infants and children have a large variety of myelinization of the brain which affects the relaxation times of brain tissue. While there is a large body of literature to be found on age averages, there is still a variation in myelination of children the same age affecting the tissue relaxation times [15, 16].

A major limitation of the presented method is an increased time demand for scanning. In this study, acquiring a dataset using the vfa method took 1:12 excluding post-processing. This is an increase in scan time of one-quarter. Adding this label/control pair which does not contribute to the final image can cause several issues. Apart from an increased overall scan time, longer scans are more prone to movement artifacts due to patients not being able to lie still for prolonged times. Furthermore, the clinical acceptance can be reduced as additional scan time is counterproductive in routine imaging. However, investing this time to reduce label/control pairs of the ASL scan might be an option. Yet, this would mean at least removing more repetitions than needed for optimization while maintaining the same image quality or obtaining the same image quality at the same scan time. Arguably, adding one label/control pair is less of a time-constraint in 2D imaging as compared to 3D acquisitions. While this study was only conducted on a low number of healthy volunteers, the results show differences by adapting the background suppression pulse timing and it can be expected that this is not different to patients. Future optimization could include an approach similar to that presented by Huber et al [10] including the presented T1 mapping procedure into the M0 scan.

To conclude, individualizing the background suppression pulse timing appears to improve the image quality of ASL scans, but further improvements are needed to avoid any negative effects of this method such as an increased time demand for scanning.

## Abbreviations

ASL: Arterial spin labeling
FA: Flip angle
MOLLI: Modified Look-Locker imaging
PCASL: Pseudo-continuous arterial spin labeling
PLD: Post labeling delay
RF: Radio frequency
vfa: Variable flip angle

## Declarations

### Guarantor

The scientific guarantor of this publication is Prof. Jens Fiehler.

### Statistics and biometry

No complex statistical methods were necessary for this paper.

### Methodology

• prospective

• experimental

• performed at one institution
